# Effects of Iron Concentration Level in Extracting Solutions from Contaminated Soils on the Determination of Zinc by Flame Atomic Absorption Spectrometry with Two Background Correctors

**DOI:** 10.1155/2012/512709

**Published:** 2012-01-12

**Authors:** Christophe Waterlot, Aurélie Pelfrêne, Francis Douay

**Affiliations:** ^1^Université Lille Nord de France, 59044 Lille, France; ^2^Groupe ISA, Equipe Sols et Environnement, Laboratoire Génie Civil et géo-Environnement (LGCgE) Lille, Nord de France (EA 4515), 48 boulevard Vauban, 59046 Lille Cedex, France

## Abstract

Zinc and iron concentrations were determined after digestion, water, and three-step sequential extractions of contaminated soils. Analyses were carried out using flame absorption spectrometry with two background correctors: a deuterium lamp used as the continuum light source (D_2_ method) and the high-speed self-reversal method (HSSR method). Regarding the preliminary results obtained with synthetic solutions, the D_2_ method often emerged as an unsuitable configuration for compensating iron spectral interferences. In contrast, the HSSR method appeared as a convenient and powerful configuration and was tested for the determination of zinc in contaminated soils containing high amounts of iron. Simple, fast, and interference-free method, the HSSR method allows zinc determination at the ppb level in the presence of large amounts of iron with high stability, sensitivity, and reproducibility of results. Therefore, the HSSR method is described here as a promising approach for monitoring zinc concentrations in various iron-containing samples without any pretreatment.

## 1. Introduction

Flame atomic absorption spectrometry (FAAS) is often described as a fast, simple, and sensitive technique for the measurement of zinc (Zn). This technique is widely used for the determination of Zn at trace levels in biological samples and food after mineralization process [1–4], but it usually requires pretreatment of samples such as sorption and/or preconcentration [5, 6], coprecipitation [7–9], liquid-liquid extraction [9, 10], solid-phase extraction [9–15], and more recently cloud point extraction [16, 17] in order to eliminate the matrix effects. However, these methods usually suffer from a lack of automation, increasing greatly the handling effort. Many research efforts have recently focused on a minimally time-consuming sample pretreatment, and consequently flow injection analysis (FIA) systems have been developed for the determination of Zn using FAAS [18–20]. Despite of these attempts, it is worth noting that the digestion process of soil samples is needed to convert them into solutions to be introduced through nebulization chambers of the FAAS.

Of the two atomic absorption lines of Zn, at 213.856 and 307.950 nm, the latter is hardly used for trace Zn analysis in FAAS because its sensitivity is 3,000 times lower than the former. Because this analytical line is subjected to spectral interferences by line overlapping at 213.859 nm and 213.853 nm due to the presence of Fe and Cu, respectively [21, 22], the determination of Zn at trace levels in the presence of Fe and/or Cu may be difficult with FAAS. For instance, the iron line at 213.859 nm cannot be completely resolved from the zinc resonance line at 213.856 nm by the optical system, resulting in a spectral interference using the conventional FAAS, without or with background correction, like deuterium lamp or Zeeman effect [23]. According to our knowledge, only the combination of the high-resolution continuum-source atomic absorption spectrometry with the least-squares background correction is able to resolve completely the problem of spectral interferences by direct overlap of absorption lines such as Fe and Zn [22, 23].

While very few studies have focused on the elimination of Fe in the determination of Zn using FAAS [24–27], the determination of Zn concentrations within the extracting solutions from contaminated soils without any pretreatment using FAAS is poorly documented, probably due to the low level of Fe and Cu matrix and the high level of Zn. Therefore, the present study focused on the use of a self-reversal background corrector. A first approach of the method has been proposed in 1983 by Smith and Hieftje [28]. A second approach has been recently developed and was called high-speed self-reversal method (HSSR method) [29]. This method was described as a universal technique covering the entire wavelength range from 190 to 900 nm. Based on previous studies in which it was shown that Cu concentrations were much lower than Zn within the contaminated soils located in the studied area [28, 29], the present work focused on the determination of Zn in extracting solutions containing high Fe concentrations using FAAS combined with the HSSR method. Results were compared with those obtained with the deuterium lamp used as the continuum-source background corrector (D_2_ method). A systematic study was first conducted in spiked Zn solutions with low and high Fe concentrations in order to handle spectral interferences and matrix effects when the mobility of Zn in contaminated soils was examined. The method was then used to detect Zn concentrations extracted by water and *aqua regia* in certified reference materials and in contaminated soils, focusing on the possible overlapping analytical line of Zn at 213.856 nm with NO molecular absorption bands in the air-acetylene flame. The HSSR method was then validated using the first three steps of the sequential extraction procedure recommended by the SM&T (standards, measurements, and testing program) using two certified reference materials and contaminated soil samples.

## 2. Materials and Methods

### 2.1. Standard Solutions and Reagents

All solutions were prepared from analytical-grade reagents unless otherwise specified. Doubly distilled water (Carlo Erba, Val de Reuil, France) was used to prepare all aqueous solutions and dilutions.

- Glacial acetic acid (Acrōs Organics, Noisy-le-Grand, France, *d* = 1.048) was used to obtain the 0.11 M acetic acid.
- 0.5 M hydroxylamine hydrochloride was prepared by dissolving 34.75 g of the solid (Acrōs Organics) in 975 mL of doubly distilled water after adding 25 mL of 2 M nitric acid (HNO_3_), prepared by diluting 12.7 mL of HNO_3_ (J.T. Baker for metal trace analysis, Deventer, Netherlands, 70%, *d* = 1.42) to 100 mL in a volumetric flask.
- 1 M ammonium acetate solution was obtained by adding 77.08 g of the solid in 1000 mL of doubly distilled water after adjusting the pH (pH = 2) with 65% HNO_3_ (*d* = 1.40).
- Hydrogen peroxide was from J.T. Baker.
- *Aqua regia* was obtained by mixing concentrated 4.5 mL of hydrochloric acid (J.T. Baker, 37%, *d* = 1.19) and 1.5 mL of HNO_3_ (70%).

All glassware and polypropylene materials were cleaned by soaking for 1 day in 0.5 M nitric acid (J.T. Baker) and by rinsing with doubly distilled water. Zinc solutions of 1, 2, and 5 mg L^−1^ in 2% HNO_3_ (Chemical Products for Analysis, Association Corporation Standard Distribution; C.P.A. groupe A.C.S.D., Voisins le Bretonneux, France) were used for the preparation of the calibration solutions and test solutions containing Fe.

### 2.2. Instrumentation

An atomic absorption spectrometer (Shimadzu AA-6800, Tokyo, Japan) with an ASC-6100 auto-sampler (Shimadzu) was used for the determination of Fe and Zn with an air-acetylene flame. For Fe, the instrumental parameters were as follows: wavelength 248.3 nm; lamp current 12 mA; bandpass 0.2 nm; a deuterium lamp (Hamamatsu, Photonics K.K., Tokyo, Japan) for the background correction. For Zn, a conventional hollow-cathode lamp from Hamamatsu was used at 213.856 nm with a 0.5-nm spectral bandpass and at 10 mA in combination with a deuterium lamp. A Zn high-intensity boosted-discharge hollow-cathode lamp from Hamamatsu was also used as the spectral radiation source. This lamp was operated with two different discharges (10 mA and 300 mA) in order to increase the emission intensity. During the analytical measurements, the Zn lamp was operated at low and high currents with a frequency of 100 Hz. At high currents (300 mA), the absorbance (Abs) measured for a narrow atomic line is low, and the specific absorption of the target element is zero as well, whereas the apparent absorbance caused by a broadband background contributor remains as high as when the lamp is operated at low current levels. In contrast, at conventional current levels (10 mA) and specific wavelength (213.856 nm), the energy related to the absorption of the target element is maximum. As a consequence, the difference in absorbance with the lamp operated at low and high currents gives the background correction.

### 2.3. Test Solutions of Zn and Fe

Test solutions containing Zn and Fe were prepared from the stock solutions described above. Samples were analyzed in pairs to allow for machine drift, for example: Zn 1 mg L^−1^; Zn 1 mg L^−1^ + Fe 0 mg L^−1^; Zn 1 mg L^−1^; Zn 1 mg L^−1^ + Fe 0.5 mg L^−1^, Zn 1 mg L^−1^; Zn 1 mg L^−1^ + Fe 1 mg L^−1^, and so forth. The experiments were conducted with all extracting solutions used (water, acetic acid, and hydroxylamine hydrochloride).

### 2.4. Soil Sampling and Pretreatment Procedure

The soil samples (agricultural fields and kitchen gardens) used in this work were collected in a contaminated area highly affected by the past atmospheric emissions of two lead and Zn smelters located in the north of France [28–32]. For each soil type, a composite sample was constituted in the ploughed layer (0–25 cm) and was prepared following the NF ISO 11464 procedure. The soil samples were air-dried at a temperature below 40°C, crushed to pass through a 2 mm stainless steel, and sieved to less than 250 *μ*m particle size with an ultracentrifugal mill (Retsch type ZM 200, Hann, Germany). Two certified reference materials, sediment (BCR-701, Piedmont, Italy) and sewage sludge-amended soil (CRM-483, Great Billings Sewage Farm, Northampton), were used for the validation method.

### 2.5. Extraction Procedures

Digestion of kitchen garden soil samples (KG1 to KG9) was carried out using a microwave oven (Berghof Speedwave MWS-2, Eningen, Germany) with a system to control the temperature inside the reactor pressure vessel. Initially, 300 mg of each sample was transferred to a 100 mL digestion tube, and a mixture of nitric acid (70%, (m/m), 1.5 mL) and hydrochloric acid (37%, (m/m), 4.5 mL) was added. After mineralization, digestion products were completed to 25 mL with doubly distilled water and stored in acid-washed plastic bottles at 4°C prior to analysis.

Water-soluble metal ions were extracted from kitchen garden soils in triplicate with the following procedure: 3 g of each representative sample was shaken with a soil/extractant ratio of 1/10 (w/v) using a rotor disc (10 rpm) for 2 h. The extract was separated from the solid residue by centrifugation (4,530 rpm, Rotanta 460 Hettich, Tuttlingen, Germany) for 20 min at room temperature. Afterwards, the solution was filtered over an acetate Millipore membrane (Millipore, 0.45 *μ*m porosity, Minisart). The solution was then placed in a polypropylene container and stored at 4°C until metal analysis.

The three-step extraction procedure was used to determine Zn fractionation in the certified and contaminated agricultural soils (A1 to A10) [33]. Each of these steps was noted as being fraction F1, F2, or F3 (Table 1).

Zinc and Fe concentrations were expressed as mg kg^−1^ dry weight (DW). For this, the moisture content of each sample was established by drying a separate 1-g sample in an oven (Binder, Tuttlingen, Germany) at 105 ± 2°C until it reached a constant mass according to the NF ISO 11465 standard.

### 2.6. Statistical Analyses

The Mann-Whitney *U* test (nonparametric statistical test) was used to find out the influence of Fe in the determination of Zn concentrations in the test solutions. For each extracting solution and background corrector, this test was carried out to determine significant differences among Zn concentrations according to the background corrector and certified or indicative values. All statistical tests were performed using Statistica 6.0 (Statsoft, Tulsa, OK, USA) for Windows. The level of significance was set at *P* < 0.05.

## 3. Results and Discussion

### 3.1. Analytical Performance and Spectroscopic Conditions

Analyses were carried out using each batch of solutions as reagent blanks. The figures of merit of both methods were established using each extracting solution [34] and are shown in Table 2.

**Figure 1:**
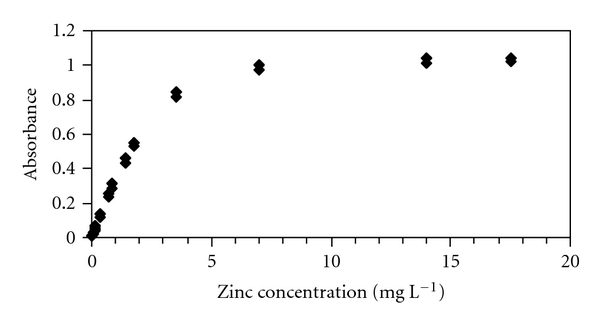
Analytical curve for Zn in 0.11 M acetic acid measured in FAAS with the HSSR method at 213.856 nm.

The calibrations covered Zn concentrations ranging from 2 to 25 *μ*g L^−1^ and were linear. The correlation coefficient ranged from 0.9983 to 0.9993, depending on the extracting solutions. In all experiments, the slopes of the calibration curves as well as the dynamic calibration ranges obtained with the HSSR method were slightly lower than those from the D_2_ method. These results indicate that the HSSR method had the lowest sensitivity and imply that the limiting absorbance values are smaller than those for the D_2_ method. As recorded for other elements [35], the cause of the calibration curve flattening is probably due to the high stray-light levels of the boosted-discharge hollow-cathode lamp. In contrast to the results obtained with the D_2_ method, no roll-over (i.e., a decrease of absorbance at high concentrations) occurred for Zn in the extracting solutions with the HSSR method (Figure 1). 

Limits of detection (LOD) were defined as the concentration equivalent to three times the reagent blank (water, acetic acid, hydroxylamine hydrochloride, ammonium acetate at pH = 2, nitric acid 14%; *n* = 10). Using the HSSR method, the LOD values were the lowest (Table 2). For instance, LOD values in F1 and F3 fractions were 3.5-fold and 3.7-fold lower than those obtained with the D_2_ method. Improvement of the LOD may be due to the reduction of the baseline noise and the better stability during the flame atomization of Zn when the HSSR method was used. The precision of this method was evaluated as the relative standard deviation (R.S.D) of 13 replicate determinations of Zn at 5 and 10 *μ*g L^−1^ and 0.5 and 1 mg L^−1^ in each extracting solution. The R.S.D ranged from 0.12 to 1.37%, reflecting a good reproducibility of the background corrector effects with solutions at low level of Zn (in the same order of magnitude of the LOD). On the other hand, under the working conditions (dilute nitric acid) no interference related to the overlapping analytical line of Zn at 213.856 nm with NO molecular absorption bands in the air-acetylene flame was detected. This result is in accordance with phenomena reported by de Oliveira et al. [36].

### 3.2. Determination of Zn in Test Solutions

**Figure 2:**
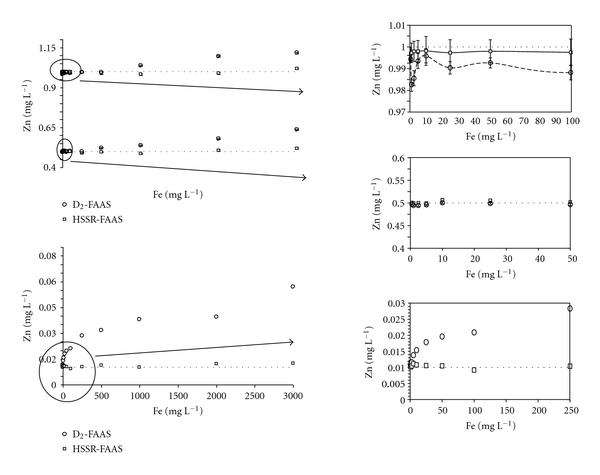
Effect of Fe on the three concentrations of Zn (0.01, 0.5, and 1 mg L^−1^) measured in water by FAAS at 213.856 nm with the D_2_ and HSSR methods.

**Figure 3:**
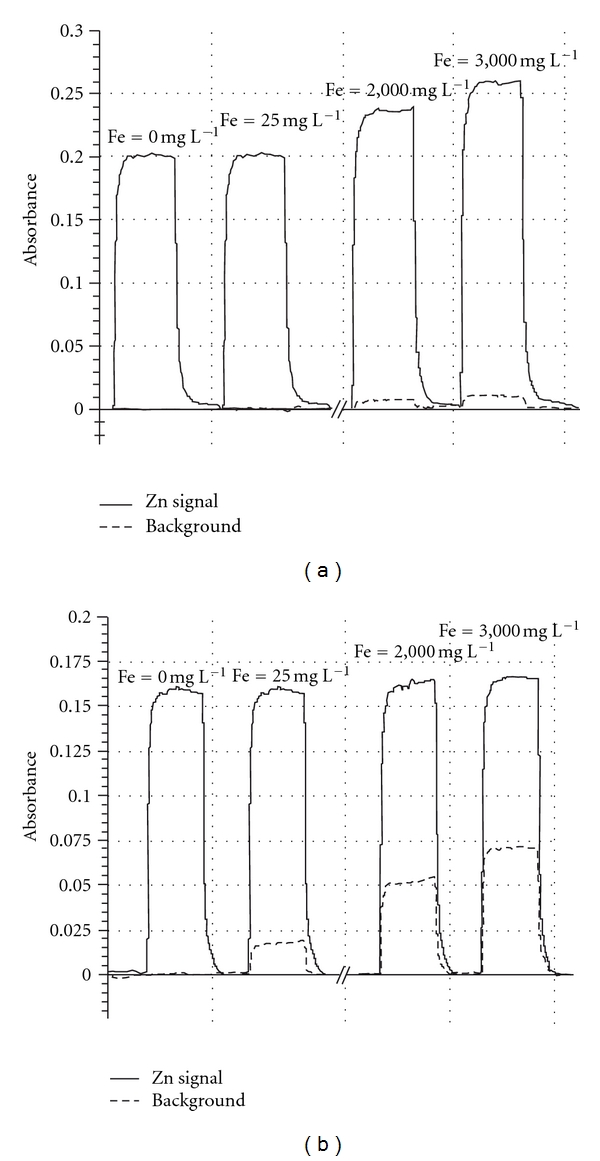
Effect of Fe in water on the signal of Zn at 0.5 mg L^−1^ using (a) deuterium background correction (D_2_ method) (b) high-speed self-reversal background correction (HSSR method).

The effects of Fe on Zn concentrations in water were studied with the two configurations selected for background correction (Figure 2). An overestimation of Zn concentrations measured in spiked solutions with the D_2_ method was observed for Zn at 0.01 mg L^−1^, Zn at 0.5 mg L^−1^ and Fe > 250 mg L^−1^, and Zn at 1 mg L^−1^ and Fe > 500 mg L^−1^. Surprisingly, Zn concentrations in these solutions with Fe concentrations ranging from 0.5 to 100 mg L^−1^ were lower than 1 mg L^−1^, reflecting an overcompensation of the deuterium background correction. In contrast, Fe concentrations below 25 mg L^−1^ did not cause any interference for deuterium corrector in the solution of Zn at 0.5 mg L^−1^ (Figures 2 and 3). These results show that the under- and overcompensation of Zn concentrations using the deuterium corrector depended on Zn concentrations and Fe/Zn ratios. 

**Figure 4:**
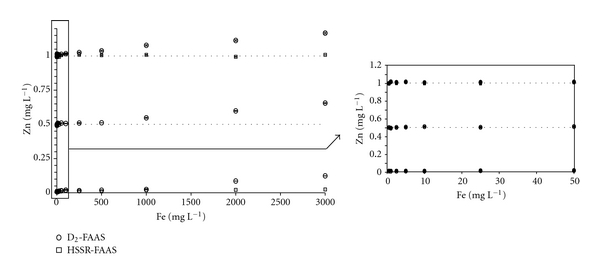
Influence of Fe on the three concentrations of Zn (0.01, 0.5, and 1 mg L^−1^) measured in 0.11 M acetic acid by FAAS at 213.856 nm with the D_2_ and HSSR methods.

**Figure 5:**
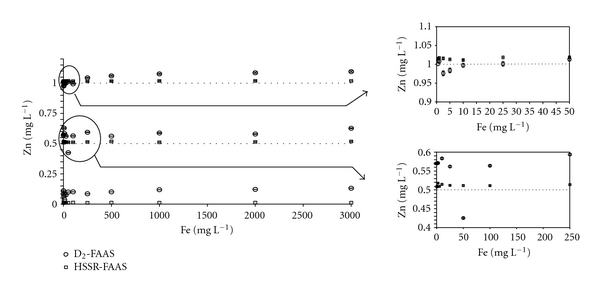
Influence of Fe on the three concentrations of Zn (0.01, 0.5, and 1 mg L^−1^) measured in 0.5 M hydroxylamine hydrochloride by FAAS at 213.856 nm with the D_2_ and HSSR methods.

While serious interferences can be observed with the D_2_ method due to the low background correction (Figure 3(a)), the presence of Fe did not show a significant effect on Zn absorbance using the HSSR method (Figure 3(b)). As shown in Figures 2 and 3, experimental Zn concentration values were very close to the theoretical ones whatever the Fe concentration within the solutions. For instance, in the mixture of Zn and Fe at 0.01 mg L^−1^ and 3,000 mg L^−1^, respectively, the mean concentration of Zn measured using the HSSR method was 0.011 ± 0.001 mg L^−1^, reflecting the efficiency of this method to eliminate the Fe spectral interference in the course of Zn determination. Similar results were obtained in 0.11 M acetic acid-spiked solutions (Figure 4). However, the increase in the signal absorbance of Zn at low concentrations in the acid solutions was lower than that in the water, showing an effect of the solvent on the concentration measured with the D_2_ method. In contrast, even at low Fe concentrations, this background correction appeared to be unsuitable for the determination of Zn in 0.5 M hydroxylamine hydrochloride solution, which is included in the SM&T program (Figure 5). Signal absorbance generally increases with the Fe concentration, leading to higher measured concentrations of Zn than those present in the solutions. Additionally, the Zn signal decreased to such an extent that it turned out to be lower than that related to the Zn concentration at 0.5 mg L^−1^ indicating either an overcorrection due to the presence of Fe and/or a suppressive effect of chloride ions on the signal of Zn [37].

For each Zn concentration, interference effects of Fe were minimized and even avoided when a boosted hollow-cathode lamp was employed for the HSSR background correction. Moreover, the Mann-Whitney *U* test results showed that Fe interferences were not significant when the Fe concentration was less than 3,000 mg L^−1^ and the ratio of Fe/Zn was smaller than 300,000.

### 3.3. Application to Soil Samples

Depending on the physicochemical parameters of soils (pH, organic matter, and Fe contents), the possible migration of Zn to the depth of contaminated soils located in the studied area was considered [32]. Modeling to predict the solubility of Zn in soils is therefore very important for the management of polluted soils and industrial sites. In this way, the potential mobility and bioavailability of Zn are often evaluated using various extracting solutions including water and those used in the SM&T sequential extraction procedure. There are recurrent problems during the determination of this element by FAAS because of the large amounts of Fe in extracting solutions and the closeness (0.003 nm) between the two atomic absorption lines of Zn and Fe [26]. First of all, pseudototal Fe, Cu, and Zn concentrations in kitchen garden soil samples were determined after their microwave-assisted digestion (Table 3).

Measurements were performed by dilution of 1 : 10 *aqua regia* extracts with doubly distilled water except for KG8, for which a dilution of 1 : 20 was necessary. Zinc concentrations were in the range 0.56–2.90 mg L^−1^ and Fe concentrations ranged from 15.8 to 37.7 mg L^−1^. In almost all cases, Zn concentrations measured with the D_2_ method were lower than those determined using the HSSR method, and significant differences (*P* < 0.05) were obtained for KG2, KG3, KG4, KG7, KG8, and KG9. The mean concentration of Zn found in the BCR-483 material using the D_2_ method was significantly lower than that measured by Pueyo et al. [40]. This result was attributed to an underestimation of the Zn concentrations related to overcompensation of the deuterium background correction observed in the Zn solution at 1 mg L^−1^ spiked with Fe (Figure 2). In contrast, the values reported for both certified materials obtained with the HSSR method were very close to those found by Pueyo et al. [40] using ICP-MS with all precautions to prevent matrix interferences. As shown for the spiked Zn solutions, and compared to the certified or indicative values, the accuracy of the results can be explained by the efficiency of the HSSR method in correcting the background of Fe near the analytical line of Zn. In order to supplement this approach, the concentrations of Zn were measured using the two background correction systems in undiluted water extracts. Data are reported in Table 4. Depending on the Fe/Zn ratio (ranging from 7 to 2,481), the concentrations of Zn measured with the D_2_ method were up to 40-fold higher than those obtained with the HSSR method. The magnitude of the difference between Zn concentrations in the water extracts from the KG2 soil sample with both background correction systems is in agreement with the results obtained using the spiked Zn solution at 10 *μ*g L^−1^ with a high Fe/Zn ratio. It reflects an undercorrection of the deuterium background system, providing an overestimation of Zn concentrations. In contrast, the Zn concentrations found in the water extracting solution from KG8 were not significantly different for the two background systems. From a statistical point of view, the high standard deviation value can explain this result but it seems clear that the low value for the Fe/Zn ratio (Fe/Zn = 7.16) is a more appropriate explanation.

Analytical studies were further carried out with the extracting solutions included in the 3 steps sequential extraction procedure proposed by the standards measurements and testing program (SM&T) of the European Union. The mean concentrations of Zn found with the D_2_ and HSSR methods in the BCR-701 and BCR-483 certified materials, as well as the certified or indicative values for this analyte and Fe, are shown in Table 5. For both certified materials, significant differences (*P* < 0.05) were found between Zn concentrations measured using the D_2_ method and certified or indicative values. An exception was noted for the Zn concentration in the F3 fraction of the BCR-483 certified material. The greatest differences were observed in step 2, in which 0.5 M hydroxylamine hydrochloride was used to release the free Fe oxides [41]

 As shown in Table 5, the high concentrations of free Fe in the F2 fraction led to an overestimation of Zn concentrations, reflecting an undercompensation of the deuterium background system. In contrast, FAAS measurements combined with the HSSR method yielded values were very close to the certified or indicatives ones, showing the efficiency of the method for correcting the Fe spectral interferences. The same general trends were observed for the distribution of Zn in contaminated agricultural soil samples (Table 6). Zinc concentrations measured with both background systems in the first fraction were very close to each other. An exception was observed with the A5 sample, which presented the highest Fe concentration. In contrast, differences between the concentrations of Zn in fractions F2 and F3 were up to 35%, mainly due to the order of magnitude of Fe concentrations in these fractions (from 494 to 3,498 mg kg^−1^). Despite the high Fe concentration found in F2 of sample A10, it is important to note that the mean concentration of Zn was lower using the D_2_ method than the HSSR method. Taking into account the liquid/solid ratio and the dilution factor, the concentrations of Zn and Fe were 0.99 and 6.6 mg L^−1^, respectively. The result is consistent with the data presented in Figure 5 reflecting an underestimation of Zn at 1 mg L^−1^ in presence of Fe from 2.5 to 10 mg L^−1^ using the D_2_ method. All of these results confirm that the HSSR method is an efficient background compensation of the spectral interferences caused by Fe during Zn measurements using FAAS.

### 3.4. Efficiency of the HSSR Method

As reported by Sweileh and El-Nemma [26], the determination of Zn in the presence of high concentrations of Fe (e.g., some geological samples, meteorites, or steels) is problematic using FAAS. From our investigations, the Zn-free solution containing 0.2%, 0.3%, and 2% Fe appeared to contain 0.13, 0.14, and 0.39 mg L^−1^ of Zn, respectively. The last value is in agreement with the previously reported spectral interference of Fe in the determination of Zn [26] indicating that the deuterium background correction is not always suitable for compensating the Fe spectral interference in Zn. We determined the trace amounts of Zn in the 2% Fe solution with the proposed method. The results are summarized in Table 7. The reported values of Zn measured by FAAS with the D_2_ method were greater than the Zn concentrations added in the Fe solution. In contrast, the Zn concentrations measured by FAAS with the HSSR method were very close to the added concentrations, proving the effectiveness of the proposed technique for correcting matrix effects and spectral interferences in FAAS.

## 4. Conclusions

The extractable concentrations of Zn (*aqua regia*, water, and sequential extractions) were determined by FAAS with two background correction systems. The first was based on the well-known deuterium background correction and the second involved the use of a single Zn hollow-cathode lamp pulsed with currents ranging from 10 to 300 mA with a frequency of 100 Hz. Depending on the extracting solutions, but also on the Zn concentration and the Fe/Zn ratio, it was demonstrated that background correction with a deuterium lamp led to significant errors (e.g., over- and underestimation). In contrast, the HSSR method appeared to be a more versatile technique for the compensation of spectral interferences caused by absorption line overlapping. The proposed method allowed Zn determination at the *μ*g L^−1^ level in the presence of large concentrations of Fe with high stability and sensitivity. With a good LOD and R.S.D, this method shows promise for monitoring zinc concentrations in various Fe-containing samples without any pretreatment. 

## Figures and Tables

**Table 1:** Sequential extraction procedure (from Rauret et al. [33]).

Steps	Extracting solutions	Nominal target phases
1	Acetic acid 0.11 M	Fraction F1: exchangeable, water- and acid-soluble
2	Hydroxylamine + hydrochloric acid 0.50 M at pH 2	Fraction F2: reducible
3	Hydrogen peroxide 8.8 M, + ammonium acetate 1.0 M at pH 2	Fraction F3: oxidizable

**Table 2:** Calibration data for the determination of Zn.

Background corrector	Calibration range (**μ**g L^−1^)	Equation of calibration curves	LOD (**μ**g L^−1^)
D_2_-FAAS^a^	2–25	Abs = 0.47790 [Zn] + 0.00553 *R^2^* = 0.9991	2.8
HSSR-FAAS^a^	2–25	Abs = 0.44075 [Zn] + 0.00015 *R^2^* = 0.9988	1.5
D_2_-FAAS^b^	2–25	Abs = 0.37643 [Zn] + 0.00030 *R^2^* = 0.9993	6.5
HSSR-FAAS^b^	2–25	Abs = 0.37154 [Zn] + 0.00074 *R^2^* = 0.9992	1.9
D_2_-FAAS^c^	2–25	Abs = 0.42581 [Zn] + 0.00080 *R^2^* = 0.9992	3.8
HSSR-FAAS^c^	2–25	Abs = 0.31517 [Zn] − 0.00044 *R^2^* = 0.9991	1.6
D_2_-FAAS^d^	2–25	Abs = 0.42600 [Zn] + 0.00038 *R^2^* = 0.9973	2.6
HSSR-FAAS^d^	2–25	Abs = 0.27455 [Zn] − 0.00029 *R^2^* = 0.9981	0.7
D_2_-FAAS^e^	2–25	Abs = 0.36182 [Zn] + 0.00015 *R^2^* = 0.9987	3.1
HSSR-FAAS^e^	2–25	Abs = 0.32234 [Zn] − 0.00070 *R^2^* = 0.9993	1.9

^a^In water.

^b^In 0.11 M acetic acid.

^c^In 0.5 M hydroxylamine hydrochloride (F2).

^d^In 1 M ammonium acetate pH = 2 (F3).

^e^In HNO_3_ 14%.

**Table 3:** Pseudototal Fe, Cu, and Zn concentrations (mean value ± standard deviation) measured in certified reference soil (*n* = 3) and kitchen garden (KG) soil samples (*n* = 3).

Samples	Fe in this work (mg kg^−1^)	Cu in this work (mg kg^−1^)	Zn certified value (mg kg^−1^)	Zn with the D_2_ method (mg kg^−1^)	Zn with the HSSR method (mg kg^−1^)
BCR-483	26,642 ± 1,180^a^	403 ± 11^c^	987 ± 37^b,c^	939 ± 48	1,013 ± 59
BCR-701	36,975 ± 342^d^	43.8 ± 1.5^e^	454 ± 19^f^	474 ± 15	478 ± 7
KG1	22,496 ± 717	35.5 ± 2.3		821 ± 10	808 ± 6
KG2	24,681 ± 799	25.6 ± 1.9		513 ± 4	531 ± 7
KG3	23,628 ± 685	13.3 ± 1.3		813 ± 15	958 ± 17
KG4	18,846 ± 578	78.2 ± 3.1		867 ± 9	895 ± 1
KG5	20,026 ± 624	74.4 ± 2.8		1,052 ± 21	1,051 ± 12
KG6	24,399 ± 790	63.3 ± 2.3		1,398 ± 21	1,410 ± 24
KG7	18,851 ± 580	27.3 ± 1.1		505 ± 3	522 ± 6
KG8	27,029 ± 890	170.4 ± 5.4		4,502 ± 25	4,842 ± 54
KG9	31,440 ± 661	12.6 ± 1.0		655 ± 5	667 ± 5

^a^From Kubová et al. [38], [Fe] = 26,700 ± 480 mg kg^−1^.

^b^Indicative value from Rauret et al. [39], (*n* = 5).

^c^From Pueyo et al. [40], [Zn] = 1,026 ± 37 mg kg^−1^; [Cu] = 373 ± 14 mg kg^−1^ (*n* = 6).

^d^From Kubová et al. (2004) [38], [Fe] = 38,580 ± 220 mg kg^−1^.

^e^Certified value: [Cu] = 46.4 ± 1.8 mg kg^−1^ (*n* = 6).

^f^From Pueyo et al. [40], [Zn] = 474 ± 10 mg kg^−1^ (*n* = 6).

**Table 4:** Water-extractable Fe and Zn concentrations (mean value ± standard deviation, *n* = 3) in kitchen garden (KG) soil samples.

Samples	Fe (mg kg^−1^)	Zn with the D_2_ method (**μ**g kg^−1^)	Zn with the HSSR method (**μ**g kg^−1^)	Fe/Zn
KG1	77.4 ± 21.2	3,100 ± 400	2,450 ± 320	31.6
KG2	6.7 ± 1.4	113 ± 40	2.7 ± 0.2	2,481
KG3	5.9 ± 1.2	1,162 ± 35	844 ± 21	7.0
KG4	68.7 ± 8.7	3,974 ± 384	3,230 ± 298	21.9
KG5	5.0 ± 0.9	799 ± 90	552 ± 101	9
KG6	113.1 ± 8.3	5,798 ± 160	4,850 ± 130	23.3
KG7	35.9 ± 10.2	1,054 ± 245	675 ± 123	53.2
KG8	120.0 ± 23.5	18,224 ± 2, 210	16,764 ± 2, 215	7.1
KG9	104.9 ± 4.3	4,789 ± 114	4,206 ± 133	24.9

**Table 5:** Certified and obtained Fe and Zn concentrations in BCR-701 (*n* = 3) and BCR-483 (*n* = 3) using the BCR three-step sequential extraction procedure.

Fraction	Metal	BCR-701			BCR-483		
		Certified value	Obtained value		Indicative value^a^	Obtained value	
			D_2_ method	HSSR method		D_2_ method	HSSR method
		mean ± U	mean ± SD	mean ± SD	mean ± U	mean ± SD	mean ± SD
		(mg kg^−1^)	(mg kg^−1^)	(mg kg^−1^)	(mg kg^−1^)	(mg kg^−1^)	(mg kg^−1^)
F1	Zn	205 ± 6	238 ± 12	205 ± 13	441 ± 39	458 ± 11	442 ± 12
	Fe	71 ± 1^b^	83 ± 1		36 ± 2^b^	36 ± 2	
F2	Zn	114 ± 5	162 ± 13	123 ± 3	438 ± 56	463 ± 18	439 ± 20
	Fe	7,698 ± 106^b^	7,732 ± 109		6,691 ± 198^b^	6,520 ± 219	
F3	Zn	45.7 ± 4.0	37.8 ± 1.2	46.6 ± 1.2	37.1 ± 9.9	35.7 ± 1.2	39.2 ± 1.5
	Fe	1,097 ± 53^b^	1,195 ± 45		1,153 ± 29^b^	1,164 ± 149	

U: uncertainty (half-width of the 95% confidence interval); SD: standard deviation.

^a^From Rauret et al. [39].

^b^Indicative values from Kubová et al. [38].

**Table 6:** Fe and Zn concentrations at each step of the SM&T sequential extraction procedure.

Samples	Fractions								
	F1			F2			F3		
	Zn (mg kg^−1^)		Fe (mg kg^−1^)	Zn (mg kg^−1^)		Fe (mg kg^−1^)	Zn (mg kg^−1^)		Fe (mg kg^−1^)
	D_2_ method	HSSR method		D_2_ method	HSSR method		D_2_ method	HSSR method	
A1	46.7	50.7	3.4	60.2	54.6	1,223	27.0	20.1	494.5
A2	132.6	120.5	18.9	143.8	138.3	1,361	114.5	111.7	1,357
A3	181.3	211.1	23.4	135.6	128.6	1,187	123.5	102.8	1,225
A4	117.0	124.0	18.5	138.0	130.3	1,398	136.9	119.3	1,115
A5	619.6	565.5	67.3	288.5	265.1	1,337	1,453	1,450	4,762
A6	108.7	111.8	0.6	205.6	177.7	1,220	77.7	65.4	638.8
A7	87.5	78.5	1.5	85.0	70.1	873.6	53.3	49.5	498.2
A8	35.2	33.9	0.2	288.1	277.2	1,800	95.7	85.0	1,046
A9	72.5	71.1	0.8	155.6	146.6	2,142	59.8	47.9	930.9
A10	147.0	152.5	18.9	397.7	409.8	2,626	347.8	334.1	3,498

**Table 7:** Determination of traces of Zn in 2% Fe solution by direct aspiration-FAAS using the D_2_ and the HSSR methods.

Zn concentration added (mg L^−1^)	Zn concentration found (mg L^−1^)	
	D_2_ method	HSSR method
0.5	0.74 ± 0.07	0.54 ± 0.03
0.75	1.02 ± 0.05	0.75 ± 0.04
1	1.20 ± 0.05	0.97 ± 0.02
1.5	1.86 ± 0.01	1.49 ± 0.01

Means and standard deviations for triplicate analyses.

## References

[1] Anzano JM, Gónzalez P (2000). Determination of iron and copper in peanuts by flame atomic absorption spectrometry using acid digestion. *Microchemical Journal*.

[2] Soriano S, Netto ADP, Cassella RJ (2007). Determination of Cu, Fe, Mn and Zn by flame atomic absorption spectrometry in multivitamin/multimineral dosage forms or tablets after an acidic extraction. *Journal of Pharmaceutical and Biomedical Analysis*.

[3] Silvestre MD, Lagarda MJ, Farré R, Martínez-Costa C, Brines J (2000). Copper, iron and zinc determinations in human milk using FAAS with microwave digestion. *Food Chemistry*.

[4] Kékedy-Nagy L, Jun Y, Darvasi E, Kékedy-Nagy L (2008). Determination of zinc in vegetal tissue microsamples by platinum-wire loop in flame atomization atomic absorption spectrometry. *Journal of Biochemical and Biophysical Methods*.

[5] Taher MA (2000). Atomic absorption spectrometric determination of ultra trace amounts of zinc after preconcentration with the ion pair of 2-(5-bromo-2-pyridylazo)-5- diethylaminophenol and ammonium tetraphenylborate on microcrystalline naphthalene or by column method. *Talanta*.

[6] Giokas DL, Paleologos EK, Prodromidis MI, Karayannis MI (2002). Development of 1-(2-pyridylazo)-2-naphthol-modified polymeric membranes for the effective batch pre-concentration and determination of zinc traces with flame atomic absorption spectrometry. *Talanta*.

[7] Soylak M, Saracoglu S, Divrikli U, Elci L (2005). Coprecipitation of heavy metals with erbium hydroxide for their flame atomic absorption spectrometric determinations in environmental samples. *Talanta*.

[8] Uluozlu OD, Tuzen M, Mendil D, Soylak M (2010). Coprecipitation of trace elements with Ni^2+^/2-Nitroso-1-naphthol-4-sulfonic acid and their determination by flame atomic absorption spectrometry. *Journal of Hazardous Materials*.

[9] Komjarova I, Blust R (2006). Comparison of liquid-liquid extraction, solid-phase extraction and co-precipitation preconcentration methods for the determination of cadmium, copper, nickel, lead and zinc in seawater. *Analytica Chimica Acta*.

[10] Roldan PS, Alcântara IL, Padilha CCF, Padilha PM (2005). Determination of copper, iron, nickel and zinc in gasoline by FAAS after sorption and preconcentration on silica modified with 2-aminotiazole groups. *Fuel*.

[11] Ghaedi M, Niknam K, Shokrollahi A, Niknam E, Ghaedi H, Soylak M (2008). A solid phase extraction procedure for Fe^3+^, Cu^2+^ and Zn^2+^ ions on 2-phenyl-1H-benzo[d] imidazole loaded on Triton X-100-coated polyvinyl chloride. *Journal of Hazardous Materials*.

[12] Tuzen M, Melek E (2008). Solid-phase extraction of copper, iron and zinc ions on *Bacillus thuringiensis israelensis* loaded on Dowex optipore V-493. *Journal of Hazardous Materials*.

[13] Ghaedi M, Niknam K, Taheri K, Hossainian H, Soylak M (2010). Flame atomic absorption spectrometric determination of copper, zinc and manganese after solid-phase extraction using 2,6-dichlorophenyl-3,3-bis(indolyl)methane loaded on Amberlite XAD-16. *Food and Chemical Toxicology*.

[14] Saljooghi AS, Fatemi SJ, Afzali D (2010). Determination of trace amounts of zinc by flame atomic absorption spectrometry after separation and preconcentration onto modified natural analcime zeolite loaded 2,3,5,6-tetra(2-pyridyl)pyrazine. *Bulletin of the Chemical Society of Ethiopia*.

[15] Vellaichamy S, Palanivelu K (2011). Preconcentration and separation of copper, nickel and zinc in aqueous samples by flame atomic absorption spectrometry after column solid-phase extraction onto MWCNTs impregnated with D2EHPA-TOPO mixture. *Journal of Hazardous Materials*.

[16] Dallali N, Zahedi MM, Yamini Y (2007). Simultaneous cloud point extraction and determination of Zn, Co, Ni and Pb by flame atomic absorption spectrometry, using 2-guanidinobenzimidazole as the complexing agent. *Scientia Iranica*.

[17] Ferreira HS, Santos ACN, Portugal LA, Costa ACS, Miró M, Ferreira SLC (2008). Pre-concentration procedure for determination of copper and zinc in food samples by sequential multi-element flame atomic absorption spectrometry. *Talanta*.

[18] Zhao S, Xia X, Yu G, Yang B (1998). Simultaneous determination of iron and zinc by pH gradient construction in a flow-injection system. *Talanta*.

[19] Chen J, Teo KC (2001). Determination of cadmium, copper, lead and zinc in water samples by flame atomic absorption spectrometry after cloud point extraction. *Analytica Chimica Acta*.

[20] dos Santos WNL, Santos CMC, Ferreira SLC (2003). Application of three-variables Doehlert matrix for optimisation of an on-line pre-concentration system for zinc determination in natural water samples by flame atomic absorption spectrometry. *Microchemical Journal*.

[21] Kelly WR, Moore CB (1973). Iron spectral interference in the determination of zinc by atomic absorption spectrometry. *Analytical Chemistry*.

[22] Welz B, Becker-Ross H, Florek S, Heitmann U (2005). *High-Resolution Continuum Source AAS: The Better Way to Do Atomic Absorption Spectrometry*.

[23] Welz B, Becker-Ross H, Florek S, Heitmann U, Vale MGR (2003). High-resolution continuum-source atomic absorption spectrometry—what can we expect?. *Journal of the Brazilian Chemical Society*.

[24] Gallego M, Garcia-Vargas M, Valcárcel M (1982). Analytical applications of picolinealdehyde salicyloylhydrazone. III. Extraction and determination of zinc by atomic absorption spectrophotometry. *Microchemical Journal*.

[25] Sweileh JA, Cantwell FF (1985). Sample introduction by solvent extraction/flow injection to eliminate interferences ion atomic absorption spectroscopy. *Analytical Chemistry*.

[26] Sweileh JA, El-Nemma EM (2004). On-line elimination of spectral interference of iron matrix in the flame atomic absorption determination of zinc by anion-exchange separation. *Analytica Chimica Acta*.

[27] Mansur MB, Rocha SDF, Magalhães FS, Benedetto JDS (2008). Selective extraction of zinc(II) over iron(II) from spent hydrochloric acid pickling effluents by liquid-liquid extraction. *Journal of Hazardous Materials*.

[28] Smith SB, Hieftje GM (1983). A new background-correction method for atomic absorption spectrometry. *Applied Spectroscopy*.

[29] Oppermann U, Schram J, Felkel D (2003). Improved background compensation in atomic absorption spectrometry using the high speed self reversal method. *Spectrochimica Acta B*.

[30] Sterckeman T, Douay F, Proix N, Fourrier H, Perdrix E (2002). Assessment of the contamination of cultivated soils by eighteen trace elements around smelters in the North of France. *Water, Air, and Soil Pollution*.

[31] Douay F, Pruvot C, Roussel H (2008). Contamination of urban soils in an area of Northern France polluted by dust emissions of two smelters. *Water, Air, and Soil Pollution*.

[32] Pruvot C, Douay F, Fourrier H, Waterlot C (2006). Heavy metals in soil, crops and grass as a source of human exposure in the former mining areas. *Journal of Soils and Sediments*.

[33] Rauret G, López-Sánchez JF, Sahuquillo A (1999). Improvement of the BCR three step sequential extraction procedure prior to the certification of new sediment and soil reference materials. *Journal of Environmental Monitoring*.

[34] Žemberyová M, Barteková J, Závadská M, Šišoláková M (2007). Determination of bioavailable fractions of Zn, Cu, Ni, Pb and Cd in soils and sludges by atomic absorption spectrometry. *Talanta*.

[35] Larkins PL (1988). The effect of spectral line broadening on the shape of analytical curves obtained using pulsed hollow-cathode lamps for background correction. *Spectrochimica Acta B*.

[36] de Oliveira SR, Raposo JL, Neto JAG (2009). Fast sequential multi-element determination of Ca, Mg, K, Cu, Fe, Mn and Zn for foliar diagnosis using high-resolution continuum source flame atomic absorption spectrometry: feasibility of secondary lines, side pixel registration and least-squares background correction. *Spectrochimica Acta B*.

[37] Sarica DY, Akim D, Özden T (2002). Determination of zinc in aerosol samples by discrete nebulization flame atomic absorption spectrometry. *Turkish Journal of Chemistry*.

[38] Kubová J, Streško V, Bujdoš M, Matúš P, Medved' J (2004). Fractionation of various elements in CRMs and in polluted soils. *Analytical and Bioanalytical Chemistry*.

[39] Rauret G, López-Sánchez JF, Sahuquillo A (2000). Application of a modified BCR sequential extraction (three-step) procedure for the determination of extractable trace metal contents in a sewage sludge amended soil reference material (CRM 483), complemented by a three-year stability study of acetic acid and EDTA extractable metal content. *Journal of Environmental Monitoring*.

[40] Pueyo M, Mateu J, Rigol A, Vidal M, López-Sánchez JF, Rauret G (2008). Use of the modified BCR three-step sequential extraction procedure for the study of trace element dynamics in contaminated soils. *Environmental Pollution*.

[41] Waterlot C, François M, Dubourguier HC Speciation and distribution of heavy metals in polluted soils.

